# Supplementary material for: Socio-demographic and behavioral correlates of excess weight and its health consequences among older adults in India: Evidence from a cross-sectional study, 2017–18
Source: PLoS One. 2023 Oct 5;18(10):e0291920. doi: 10.1371/journal.pone.0291920 (PMC10553247; doi:10.1371/journal.pone.0291920)
Supplement: Attachment — Submitted filename: Response To Reviewer.pdf [file pone.0291920.s001.pdf]

Comments to the Author

1. Is the manuscript technically sound, and do the data support the conclusions?

The manuscript must describe a technically sound piece of scientific research with data that supports the conclusions. Experiments must have been conducted rigorously, with appropriate controls, replication, and sample sizes. The conclusions must be drawn appropriately based on the data presented.

Reviewer #1: Partly

Reviewer #2: Partly

---

2. Has the statistical analysis been performed appropriately and rigorously?

Reviewer #1: Yes

Reviewer #2: Yes

---

3. Have the authors made all data underlying the findings in their manuscript fully available?

The PLOS Data policy requires authors to make all data underlying the findings described in their manuscript fully available without restriction, with rare exception (please refer to the Data Availability Statement in the manuscript PDF file). The data should be provided as part of the manuscript or its supporting information, or deposited to a public repository. For example, in addition to summary statistics, the data points behind means, medians and variance measures should be available. If there are restrictions on publicly sharing data—e.g. participant privacy or use of data from a third party—those must be specified.

Reviewer #1: Yes

Reviewer #2: Yes

---

4. Is the manuscript presented in an intelligible fashion and written in standard English?

PLOS ONE does not copyedit accepted manuscripts, so the language in submitted articles must be clear, correct, and unambiguous. Any typographical or grammatical errors should be corrected at revision, so please note any specific errors here.

Reviewer #1: Yes

Reviewer #2: No

---

5. Review Comments to the Author

Please use the space provided to explain your answers to the questions above. You may also include additional comments for the author, including concerns about dual publication, research ethics, or publication ethics. (Please upload your review as an attachment if it exceeds 20,000 characters)

Reviewer #1:

1. Author should include some important interaction findings in the abstracts' results section.

Thank you for the comment. We have reported the important findings from the multivariable analysis in the abstract's results.

2. Is the LASI data available in any online repository? A link can be included.

Yes, the LASI data are available in the repository of the Gateway to Global Aging Data and the accessible link has been provided now.

3. Please do not mention "untouchables": SC and ST are specifically defined in the Indian Constitution. A reference can be added.

Done! The variable description table now includes a description of caste categories in our study. Relevant citations are provided as well.

4. The discussion should begin with the hypothesis behind the study.

Thank you for this comment. The beginning of the discussion is now with the objectives of our study.

5. Line 337: Please include a reference for this statement about "Numerous research has looked at the relationship between obesity and the illnesses mentioned above individually and has come to similar conclusions."

We appreciate your suggestion. Relevant citations are provided now.

6. Percentage mentioned in the should be in round figure as it added in the results.

Thank you for the suggestion. The percentages reported in the results are now in round figures.

7. Why authors have given crude odds for factors as already given bivariate association in previous table.

Sure thing! We have now removed the paragraph that reports the unadjusted results from the multivariable analysis in the results section.

8. Figure is not clearly visible. Authors may add new figure with high resolution.

Thank you for the suggestion! In the revised manuscript, we have provided the figures with improved quality. Please let us know if this helps.

9. What is unique about this study? The association between obesity and NCD is well known. Even obesity leads to diseases like hypertension, diabetes, CVD, and stroke. In this study, result showed a higher percentage of older adults with hypertension (30.3%), diabetes (46.3%), and CVD (41.3%) were obese. Previous research also have similar findings like person with higher socio-economic have higher likelihood of obesity.

We appreciate your comment. We also agree that the readers of the journal will look for the novelty in our study. To that end, we have revised all the sections of the manuscript and now we focus on the socioeconomic, behavioural and health-related correlates of excess weight among older Indians. There is dearth of such studies conducted among community-dwelling older Indians. Especially, the large sample size in the LASI data was a major strength of our study. These are highlighted now.

Reviewer #2:

An interesting study as its used national epidemiological data, however there is lack of focus in the contents, unclear variables definitions and discussion. Moderate alcohol intake is part of the variables in the objectives but no proper definition and description in the results/discussion. Some of the results mention in the discussion and conclusion are not from the analysis presented. There are some ambiguous descriptions which need revision. Suggest to do proper language editing prior to final submission.

We apologize for the errors. This was an oversight on our part. In the revised manuscript, we have provided the proper definitions for the selected variables including the alcohol intake. We have revised the discussion and conclusions based on our findings. We have also corrected for typos, grammar, punctuation, and sentence structuring errors.

Abstract - In the methodology, I would suggest to include the BMI level definition for obesity used in this study. In the conclusion, the statement 'The present study suggests that introducing preventative measures such...' In my opinion, the study did not assess such preventative measures, hence it is not fair to claim such measures particularly food policies and healthy diet were not part of the variables studied.

We appreciate your comment. The conclusion section is revised to connect with the current findings.

Line 94 - please revise the statement for reference 18, they used BMI equal or more than 25 to describe excess weight (overweight and obese), not obesity per se.

Thank you! The statement is modified now.

Line 129-135: Objectives - Please revise to make them consistent with results and discussion (My suggestion: focus on the three areas - sociodemographic, health profiles and health behaviours).

a) what does comprehensive prevalence means?

This was an oversight on our part, we apologise. We have removed the term “comprehensive prevalence” in the revised manuscript.

b) what do you mean by '...independent prediction'? Age is not included in the results, but mention in this objective. Education is mentioned in b) and d). Why 'education' specifically chosen as one of the focus?

Thank you for this helpful suggestion. The statements on objectives of the study are revised and corrected for these errors.

c) Moderate alcohol - not described in variable definition and not clear in the results/ discussion.

Done! The description of the measurements including that of alcohol and tobacco consumption has been provided in the revised manuscript.

Study Population
Line 159: What does '...(including Sikkim)' means?

We apologise for this error. These words (including Sikkim) are removed now in the revised manuscript.

Variable definitions - this section needs further descriptions in the text, please elaborate the operational definitions used.

Elaborate definitions of the selected variables have been added now.

Line 142 - Please use own words instead of '....' from reference 35.

We appreciate this comment. We have revised the data section and paraphrased the statements.

Line 168 - the number stated is the number of participants included in this analysis, not sample size determination.

Thanks for your comment. The statement is revised to offer more clarity.

Line 171: Ethic - please provide ethics approval statement for the LASI study. Modify last sentence in line 176.

Ethics approval statement is provided now. And the line 176 is modified to offer more clarity.

Line 172 - typo 'publicly' not 'publically'

We have corrected this typo. We appreciate your comment.

1. Line 182-186: The definition provided is the standard definition for overweight and obesity, not Asian- specific definition. Please clarify, which definition is being used for this manuscript.

The BMI categories were based on the standard definition and according to the classification of the World Health Organization. This is clarified now in the revised manuscript.

2. Sociodemographic data:
- how does rural and urban being defined in the study

We have clarified this in the table of variable description in the revised manuscript. We appreciate your suggestion.

- Caste: please explain what does ST, SC, OBC means and how are they being identified (which one is lower or higher caste?)

We appreciate your comment on caste. We have now clearly stated of caste stratification in the revised manuscript.

-Wealth: how does the wealth being defined (e.g. how do you classify poorest - any specific income level?)

We appreciate your comment on wealth classification. We have now clarified the wealth classification in the revised manuscript.

3. Disease profile:
- Hypertension: was it self reported or any blood pressure measurement done for the study population (e.g. how do participants know about mean systolic or diastolic blood pressure?)

We have clarified this in the table of variable description in the revised manuscript. We appreciate your suggestion.

- What does cardiovascular diseases consist of? How does participants know they are having cardiovascular disease? Some definition includes hypertension and stroke as cardiovascular disease, how about this study?

Thank you for the comment! The term "cardiovascular diseases" in the earlier submission included heart diseases only. To offer more clarity, the term is replaced with "heart disease" in the revised manuscript throughout.

(Table 1, line 189)

4. Health behaviours:
- How does physical activity being assessed? What does it consist of?

We have clarified this in the table of variable description in the revised manuscript. We appreciate your suggestion.

- Tobacco consumption: How is it being classified? (Confusing - third group 'smoke tobacco but do not smoking', what does it mean?

We have clarified this in the table of variable description in the revised manuscript. We appreciate your suggestion.

- Alcohol consumption: How is it being classified? Which group describes 'moderate consumption'?

We have clarified this in the table of variable description in the revised manuscript. We appreciate your suggestion.

-How does self rated health being assessed, was it only two options of 'good' and 'poor' were given to participants

We have clarified this in the table of variable description in the revised manuscript. We appreciate your suggestion.

Statistical Analysis:
Not clear the importance of stating the equation for logistic regression.

Thank you for the comment. The equation for logistic regression is removed now.

There is a need for clear description the meaning of Model 1 and model 2 for regression, how do you select which variables for multiple logistic regression.

We appreciate your comment. As such, in the statistical analysis section, we have provided more description of models and the control variables.

Results:
Line 227 and Table 3, please be consistent with the topic, why does it suddenly change to non-communicable diseases.

This was an oversight on our part, we apologize. The titles of the tables and the description have been revised and made consistent throughout. We report socio-economic, behavioural and health-related factors associated with obesity, as mentioned in the title of our study.

Line 255 onwards - suggest to focus, simplify and describe the important findings from the results. What does the results in Table 4 means? Instead of repetitively writing back the findings in the Table 4 in the text.

Sure thing! The results section has been revised and shortened as per suggestion.

Please be careful with the interpretation of the regression analysis, few statements of 'more obese' seem inappropriate (line 258, 260, 268). MPCE quintile - I am not sure what does this means.

Thank you for the comment. The results section is revised extensively and corrected for these errors. A description of monthly per capita consumption expenditure (MPCE) quintile has been provided in the methods section.

Suggest to focus discussed the results with adjusted OR, instead of both. Line 275 - wrong result for SRH.
Suggest to focus to the study objectives.

We appreciate your suggestions, thank you. The reported results with unadjusted ORs are removed now and the results section now focuses on the adjusted estimates. The result with adjusted OR of SRH is also provided now.

Discussion - please highlight the areas related to the study objectives

The discussion is revised and it now focuses on the main findings of our study.

Limitation - Confusing, particularly second, third and fourth

Thank you! The limitations have been revised in the updated version with detailed explanation.

Conclusion is confusing, not consistent with the study objectives - suggest to revise, focus to the study findings and clearly state recommendations. Nutrition is not being assessed but included in the conclusion as part of findings?

We appreciate your suggestion. The conclusion part is significantly revised to focus on the study findings and provide clear implications.

line 382 - 'age' was not reported in the study result/ analysis, but described as a factor for obesity.

Thank you for pointing this out! The conclusion is revised accordingly and removed mentioning age, and the need for age-stratified analysis has been mentioned in the limitations.

Reference 35 - please follow proper suggested citation:
International Institute for Population Sciences (IIPS), National Programme for Health Care of Elderly (NPHCE), MoHFW, Harvard T. H. Chan School of Public Health (HSPH) and the University of Southern California (USC) 2020.

Longitudinal Ageing Study in India (LASI) Wave 1, 2017-18, India Report, International Institute for Population Sciences, Mumbai.

Thank you for taking your time to review our manuscript. The references have been updated now.

We are grateful for your comments and suggestions.
