# Supplementary material for: Socio-demographic and behavioral correlates of excess weight and its health consequences among older adults in India: Evidence from a cross-sectional study, 2017–18
Source: PLoS One. 2023 Oct 5;18(10):e0291920. doi: 10.1371/journal.pone.0291920 (PMC10553247; doi:10.1371/journal.pone.0291920)
Supplement: Attachment — Submitted filename: Revised_Manuscript_with Editors Comments.docx [file pone.0291920.s002.docx]

**Socio-demographic, behavioral and health-related correlates of excess weight among older adults in India: Evidence from a cross-sectional study, 2017-18**

**Amiya Saha ^1^, T. Muhammad^1^, Bittu Mandal ^2^, Mihir Adhikary^1^ , Papai Barman ^1^**

**^1^** Department of Family & Generations, International Institute for Population Sciences, Mumbai, India

**^2^** School of Humanities and Social Sciences, Indian Institute of Technology, Indore, Indore, India

*** Corresponding author**

**Amiya Saha**

Email: [amiyasaha4444@gmail.com](mailto:pawankumaryadavbhu@gmail.com)

ORC ID: [0000-0001-8703-8623](https://orcid.org/0000-0001-8703-8623)

**Abstract**

**Background**

Rapid population aging is expected to become one of the major demographic transitions in the twenty-first century due to the continued decline in fertility and rise in life expectancy. Such a rise in the aged population is associated with increasing non-communicable diseases. India has suffered from obesity epidemic, with morbid obesity affecting 5% of the population and continuing an upward trend in other developing countries. This study estimates the prevalence of excess weight among older adults in India and assesses its regional variations along with socio-demographic and health-related factors.

**Methods**

The study used data from the Longitudinal Ageing Study in India (LASI) wave 1 (2017–18). A total sample of 25,952 older adults (≥ 60 years) was selected for the study. Descriptive statistics, bivariate Chi-Square test, and logistic regression models were applied to accomplish the study objectives. Body mass index (BMI) has been computed for the study according to the classification of the World Health Organization.

**Results**

Overall, 23% of the older adults (≥ 60 years) were estimated with excess weight in India, which was higher among women irrespective of socioeconomic and health conditions. The highest prevalence of excess weight (≥33.2 %) was observed among older adults in states like Kerala, Karnataka, Himachal Pradesh, Punjab, and Sikkim. After adjusting for selected covariates, the odds of excess weight were higher among females than males [OR: 2.17, 95% CI: 1.86-2.54]. Similarly, the likelihood of excess weight was 1.93 times higher among the respondents who were living in urban areas compared to their rural counterparts [OR: 1.93; 95% CI: 1.69,2.21]. Higher level of education has a significant positive association with the likelihood of excess weight. Similarly, older adults who belonged to the richest strata [OR: 1.77, CI: 1.46, 2.15] of the household wealth index had a significantly higher likelihood of excess weight than older adults of the poorest strata. Having hypertension, diabetes and heart diseases were associated with excess weight among older adults. Regional variations were also observed in the prevalence of excess weight among older adults.

**Conclusion**

The findings suggest that introducing measures that help reduce the risk of non-communicable diseases, and campaigns to encourage physical activity, and community awareness may help reduce the high burden of excess weight and obesity among older Indians. The findings are important for identifying the at-risk sub-populations and for the better functioning of any public health programme and suitable intervention techniques to lower the prevalence and risk factors for excess weight in later life.

**Keyword**s

Excess weight, LASI, Socioeconomic and Health status, Older adults, Determinants

**Background**

Rapid population aging is expected as a result of the major demographic transitions in the twenty-first century due to the continued decline in fertility and rise in life expectancy [1]. By 2100, there will be 3.1 billion people over the age of sixty, which is a threefold increase from 2017 [2]. Asian countries have a higher rate of population aging and a higher absolute number of older persons, despite the relative share of the older population being higher in Western countries [3]. About 104 million people in India are 60 or older, constituting 8.6% of the total population. By 2050, the percentage is expected to rise to 20% of the population [4].

An increase in noncommunicable diseases is associated with population aging and increased life expectancies [5]. A significant rise in morbidities is brought on by obesity, which is a leading lifestyle disease worldwide [6] and has recently grown to be a major global public health concern [7,8]. It is considered the main factor contributing to the onset and severity of noncommunicable diseases [9]; obesity also raises mortality risks and affects the quality-of-life years [10]. For the past three decades, advanced regions like the USA and Europe have suffered from a severe obesity problem [11] and it was also reported that the majority of the world's population lives in countries where issues associated with obesity affect more individuals than those who are underweight [12]. The World Health Organization (WHO) reports the prevalence of obesity has tripled globally between 1975 and 2016 [13]. The prevalence of obesity, traditionally thought to be a concern in developed countries, is becoming a major public health challenge in low- and middle-income countries [14]. In 2019, 5.02 million people died prematurely owing to obesity, nearly six times as many as from HIV/AIDS, according to the Global Burden of Disease (GBD) study [15]. Over 8% of all deaths globally in 2019 were related to obesity; the figure was merely 4% in 1990 [16].

India also suffered from obesity epidemic, with morbid obesity affecting 5% of the population in the twenty-first century and is continuing an upward trend seen in other developing countries [17]. It has also seen a greater increase in the prevalence of obesity than the global average. An Indian study of older adults found that the prevalence of excess weight (≥25.0 kg/m^2^) was 14% in 2007 [18]. These could be different from younger population groups due to changes in body composition, height, food intake, and energy expenditure that occur as people age [19,20] and they can be prevented through behavioral and lifestyle changes [21]. Communities and environments supporting healthy lifestyle choices are essential in people's perception [21]. In addition to having more body fat, older persons also have altered distributions of that fat; similarly, aging is associated with loss of height and mass [22]. The average calorie intake and hunger level in older adults are often lower. Furthermore, as people age, their level of physical activity declines [22]. Due to their frailty, sickness, and impending death, older adults usually lose weight over time [22]. Studies from developed countries reveal that obesity may negatively affect morbidity more than mortality in later life [23, 24]. Previous studies have also found associations between obesity, depression [25] and diminished quality of life [26] among older adults.

Although more information is available on the physical, social, and economic factors that are associated with higher body mass index (BMI) scores in younger people [24], there is a dearth of knowledge on how patterns of obesity differ across different segments of the older population [27]. Himes [28] found that older women are more likely to be overweight and obese than older males, according to data from the Assets and Health Dynamics of the Oldest Old Survey and the Longitudinal Study of Aging. As documented, females, better socioeconomic status, and living in an urban area are important socio-demographic factors associated with higher BMI levels [29], and the behavioural and health factors include physical inactivity, smoking, self-rated health, exposure to noncommunicable diseases such as hypertension, heart disease and diabetes [29]. Studies have also reported that depressive symptoms [30], social discrimination [31] and loneliness [32] have negative effects on excess weight and, are further associated with increased healthcare costs [33] among older adults. Due to the fact that aging is convoyed by a decline in physical activity and an increase in sedentary lifestyles, older adults in low- and middle-income groups are particularly susceptible to obesity [34].

This study aimed to a) assess the prevalence of excess weight among community-dwelling older adults in India and its states; and to b) determine the socio-demographic, behavioral, and health-related factors of excess weight including gender, age, education, marital status, smoking, health status and place of residence. Understanding the prevalence of excess weight and its risk factors in older adults is necessary to develop effective clinical and community interventions to reduce the morbidity and mortality related to excess weight.

**Methods**

**Data source**

The Longitudinal Aging Study in India (LASI) wave 1 (2017–18), a national and state-representative survey of aging and health, provided the data for the current study. In its initial round, the LASI surveyed 72,250 samples of adults 45 and over throughout all 35 Indian states and union territories (UT) [35]. The major goal of the LASI survey was to offer longitudinal, valid, and reliable information on the socioeconomic and health status of the older Indian population. The LASI employed a multistage stratified area probability cluster sampling design to determine the final units of observation. LASI employed a three-stage sample design in rural areas, while in urban areas, it employed a four-stage sample design. Primary Sampling Units (PSUs) were chosen in each state and UT in the first stage. In the second stage, villages in rural regions and wards in urban areas were chosen in the selected PSUs. In the third round, households in selected villages were selected in rural areas. Urban sampling, however, required an additional step. One Census Enumeration Block (CEB) was specifically chosen at random in each urban region during the third stage. From this CEB, households were chosen as a fourth stage. The survey report included the complete methodology, including all details on the survey's design and data collection [35].

**Study population**

The current study used secondary data, specifically LASI Wave 1, which has a total sample of 73,396 adults aged 45 years and older and their spouses, regardless of age, with no missing values in age reporting. The respondents were reached out at their houses during the face-to-face interviews [35]. In this study, the participants were older adults, 60 years of age and above, who provided detailed information on their biometric measurements. After removing respondents less than 60 years (n = 37,924), those who had incomplete information on BMI (n = 532), and those who also provided incomplete information on other factors associated with excess weight (n = 2,451), the number of participants in this study included 25,952 older adults. **Figure 1** shows the inclusion and exclusion criteria for the study sample.

**Figure 1 Selection criteria of the sample study**

**Ethics statement**

The study is based on publicly available data (<https://g2aging.org/>), and survey organizations that carried out the field survey for the purpose of data collection also obtained the respondent's prior agreement. Ethics approval was obtained from the Central Ethics Committee on Human Research (CECHR) under the Indian Council of Medical Research (ICMR) and the Institutional Review Boards of collaborating organizations including the International Institute for Population Sciences (IIPS), Mumbai and the Ministry of Health and Family Welfare, Government of India. All processes related to the survey were carried out in accordance with the relevant guidelines and regulations of ICMR. With support from the Ministry of Health and Family Welfare (MoHFW), the International Institute for Population Sciences (IIPS), the United Nations Population Fund (UNPF), and other organisations, the LASI was completed.

**Variable description**

**Outcome variable**

The outcome of interest i.e. body mass index (BMI) was measured based on height and weight of older respondents. “Height and weight of adults were measured using the Seca 803 digital scale” [35]. It was categorized according to the classification of the World Health Organization: underweight (<18.5 kg/m^2^), normal weight (18.5–24.9 kg/m^2^), overweight (25.0–29.9kg/m^2^), obesity (≥30.0 kg/m^2^) [36]. It was further coded as 0 “no-excess weight” if the respondents had a score of BMI **≤** 24.9 kg/m^2^ and “excess weight” as 1 if the respondents had a score of BMI ≥ 25.0 kg/m^2^ [37].

**Predictor variables**

The study included three sets of determinant variables: (1) socio-demographic; (2) behavioral; and (3) health-related factors. A detailed description of the predictor variables appears in **Table 1**.

| Table 1 Description of the explanatory factors included in the study, Longitudinal Aging Study (LASI) Wave 1, India 2017-18 | | | | | | | |
| --- | --- | --- | --- | --- | --- | --- | --- |
| Socio-demographic | | | | | | |  |
| Variables | **Categories** | | | **Description of the category** | | | |
| Sex | | Male | | | | Sex of the respondent was available in male-female categories. | |
|  |  | Female | | | |  |  |
| Place of residence | | Rural | | | | Place of residence (rural/urban) was determined according to the administrative division of India followed in Census of India, 2011. Households in urban areas included those in towns, wards and Census Enumeration Blocks whereas, households in rural areas include those in villages (size varies from 0-10,000 population [35]. | |
|  |  | Urban | | | |  |  |
| Religion | | Hindu | | | | Religion was categorized into Hindu, Muslim, Christian, and others [38]. | |
|  |  | Muslim | | | |  |  |
|  |  | Christian | | | |  |  |
|  |  | Others | | | |  |  |
| Caste | | Scheduled castes (SC) | | | | Caste was coded as Scheduled castes (SC), Scheduled Tribes (ST), Other Backward Class (OBC) and others. SC and ST are India's most economically and socially disadvantaged groups. According to the Hindu caste system, the ST contains a segment of the population that is socially isolated and has a low economic position. People who were "educationally, economically, and socially backward" are classified as OBC. In the old caste order, the OBC is seen as being at the bottom yet somewhat above the most disadvantaged communities. The “other” caste category is identified as those having higher social standigs [39, 40]. | |
|  |  | Scheduled Tribes (ST) | | | |  |  |
|  |  | Other backward classes (OBC) | | | |  |  |
|  |  | Others | | | |  |  |
| Education | | No education | | | | There were four categories for educational status: no education, up to primary, up to secondary and secondary and above. | |
|  |  | Up to primary | | | |  |  |
|  |  | Up to secondary | | | |  |  |
|  |  | Secondary and above | | | |  |  |
| Marital status | | Currently in union | | | | Our study has categorized marital status into binary, “Currently in union” those who responded as “currently married” and all other categories as “Currently not in union” including those who responded as widowed, never married, separated, divorced, and deserted [41]. | |
|  |  | Currently not in union | | | |  |  |
| Wealth | | Lowest | | | | Using information on household consumption data, the monthly per capita consumption expenditure (MPCE) quintile has been assessed. The sample households were surveyed using sets of 11 and 29 questions on spending on food and non-food items, respectively. Food expenditures were collected during the seven-day reference period, whilst non-food expenditures were collected over 30-day and 365-day reference periods. Using 30-day reference period, expenses for both food and non-food items were standardised. The MPCE is calculated and used as a summary indicator of consumption. The variable was further divided into five quintiles, i.e., from lowest to highest [35]. | |
|  |  | Lower | | | |  |  |
|  |  | Middle | | | |  |  |
|  |  | Higher | | | |  |  |
|  |  | Highest | | | |  |  |
| Regions | | North | | | | The regions were coded as North, West, Northeast, East, Central, and South. | |
|  |  | West | | | |  |  |
|  |  | Northeast | | | |  |  |
|  |  | East | | | |  |  |
|  |  | Central | | | |  |  |
|  |  | South | | | |  |  |
| Behavioural \| \|; Physical activity \| Frequent \| There were three categories for physical activity categories: regular (every day), rare (more than once a week, once a week, once to three times a month), and never. Physical activity was assessed using the following question: “How often do you take part in sports or vigorous activities, such as running or jogging, swimming, going to a health centre or gym, cycling, or digging with a spade or shovel, heavy lifting, chopping, farm work, fast bicycling, cycling with loads”? [35].; \| Rare \|; \| Never \|; Consumption of tobacco \| Never consumed tobacco \| Consumption of tobacco was categorised by asking three questions to the redondents during survey; (i) Have you ever smoked tobacco (cigarette, bidi, cigar, hookah, cheroot) or used smokeless tobacco (such as chewing tobacco, gutka, pan masala, etc.)?.” Those who respond no was coded as “never consumed tobacco.” (ii)“What type of tobacco product have you used or consumed?.” Those who repond Smokeless tobacco (such as chewing tobacco, gutka, pan masala, etc.) was coded as “Currently consumed smokeless tobacco” and both Smoke and smokeless tobacco was coded as “Consumed both smoking and smokeless tobacco.” (iii) "Do you currently smoke any tobacco products (cigarettes, bidis, cigars, hookah, cheroot, etc.)?.” Those who respond yes was coded as “currently smoking” [35].; \| Currently smoking \|; \| Currently consumed smokeless tobacco \|; \| Consumed both smoking and smokeless tobacco \|; Consumption of alcohol \| Never consumed alcohol \| Similarly, consumption of alcohol was categorised by asking three questions to the redondents during survey; (i) Have you ever consumed any alcoholic beverages such as beer, wine, liquor, country liquor etc.?.” (ii) “In the past three months, on an average, how frequently [on how many days], have you had at least one alcoholic drink? (For example, beer, wine, or any drink, such as country liquor, containing alcohol.?.” (iii) "In the last 3 months, how frequently on average, have you had at least 5 or more drinks on one occasion??.” Those who respond no was coded as “never consumed alcohol.” Consumed none, less than once a month in past three months was coded as “frequently consumed but not a heavy drinker”; those who drank one to four times a week, one to four times a day, or five or more times a day but did not drink more than five drinks at once in the previous 30 days was coded as “Infrequently consumed but not a heavy drinker” and those who, at least once during the previous 30 days, consumed five or more  alcoholic beverages was coded as “heavy drinker” [35].; \| Frequently consumed but not a heavy drinker \|; \| Infrequently consumed but not a heavy drinker \|; \| Heavy drinker \| Health-related | | | | | | | |
| Hypertension | | Yes | | | | Hypertension was defined based on measured and self-reported information, as i. those who had a mean systolic blood pressure ≥140 mmHG and/ or mean diastolic blood pressure ≥90 mmHG (based on the last two averaged of three readings) [42]; ii. ever diagnosed with hypertension by any health professionals (yes and no) [35] and iii. taking any medications or under any diet restrictions (yes and no) [35]. If the respondents fall into any of these three, then they were considered as “having hypertension” and others were categorised as “no.” | |
|  |  | No | | | |  |  |
| Diabetes | | Yes | | | | Diabetes was sourced from the question asked during the survey “Has any health professional ever told you that you have diabetes?.” Respondents were asked to respond yes and no [35]. Those who reported “yes” were classified as diebetic and otherwise no. | |
|  |  | No | | | |  |  |
| Heart disease | | Yes | | | | Respondents were asked “Has any health professional ever told you that you have heart disease such as Coronary heart disease?.” Respondents were asked to respond yes and no [35]. Those who reported “yes” were classified as having heart disease and otherwise no. | |
|  |  | No | | | |  |  |
| Stroke | | Yes | | | | Similarly stroke was also sourced by asking the question during survey “Has any health professional ever told you that you have Stroke?.” Further, respondents were asked to respond yes and no [35]. Those who reported “yes” were classified as having diagnosed with stroke and otherwise no. | |
|  |  | No | | | |  |  |
| Self-rated health (SRH) | | | Good | | Health status was assessed by self-reported health (SRH) [35]. In the survey, individuals were asked to rate their health between 1 to 5, where 1 denoted very good, and 5 denoted very bad. Thus 1 to 3 score (very good, good and fair) was coded as good, and 4 to 5 (poor and very poor) was coded as bad [41]. | | |
|  |  |  | poor | |  |  |  |

**Statistical analysis**

Descriptive statistics and bivariate analysis were used to evaluate the prevalence of excess weight by socioeconomic status and health and behavioural factors. The significance level of the bivariate associations were determined using Chi-Squared tests. In addition, binary logistic regression analysis was used to examine the association between different socioeconomic and health-related factors and excess weight among older adults.

This study further examined the possible determinants of excess weight. Model 1 provides the univariate association of excess weight with the socioeconomic, behavioral and health characteristics of older adults. Model 2 (full model) was controlled for all the selected covariates in this study and provides the adjusted associations of excess weight with the socioeconomic, behavioural and health characteristics of older adults. The results are presented in the form of crude and adjusted odds ratios (OR) along with 95% confidence intervals, and standardised beta coefficients. All the statistical analysis was performed using STATA version 16.0 (Stata Corp, LP, college station, Texas), and ArcGIS 10.8 software for the state-level mapping.

**Results**

**Socioeconomic and demographic profile of older adults**

**Table 2** presents the socioeconomic and demographic proﬁle of older adults. Around 23% of the older adults had excess weight. More than 55% of participants were women. Almost two third of the respondents had no education, and nearly 38% of older adults were not in a marital union. Around 22% of the respondents belonged to the lowest stratum of household wealth. Around 68% of the respondents never did physical activity. Around 13% of the older adults were current smokers, nearly 21% of the respondents consumed smokeless tobacco, while 3% of the respondents consumed both. Moreover, 6%, 13% and around 1% respondents were frequent, infrequent, and heavy alcohol drinker, respectively. More than half of the older adults reported being diagnosed with hypertension, while 14% and 5% older adults had diabetes and heart diseases, respectively. More than 23% of the older adults reported poor self-rated health at the time of survey.

| Table 2 Characteristics of the study sample of older adults (60 years and above) in India 2017-18 | | |
| --- | --- | --- |
| Background | **Sample (N)** | **Percentage (%)** |
| Excess weight |  |  |
| No | 20,056 | 77.28 |
| Yes | 5,896 | 22.72 |
| Sex |  |  |
| Male | 11,653 | 44.9 |
| Female | 14,299 | 55.1 |
| Place of residence |  |  |
| Rural | 18,473 | 71.19 |
| Urban | 7,478 | 28.81 |
| Religion |  |  |
| Hindu | 21,494 | 82.82 |
| Muslim | 2,863 | 11.03 |
| Christian | 642 | 2.47 |
| Others | 952 | 3.67 |
| Caste |  |  |
| Schedule caste | 4,957 | 19.1 |
| Schedule tribe | 1,916 | 7.38 |
| Other backward class | 11,849 | 45.66 |
| Others | 7,229 | 27.86 |
| Education |  |  |
| No education | 17,774 | 68.49 |
| Up to primary | 2,877 | 11.08 |
| Up to secondary | 1,745 | 6.72 |
| Secondary & above | 3,556 | 13.7 |
| Marital status |  |  |
| Currently in union | 16,075 | 61.94 |
| Currently not in union | 9,877 | 38.06 |
| MPCE quintile |  |  |
| Lowest | 5,599 | 21.57 |
| Lower | 5,616 | 21.64 |
| Middle | 5,421 | 20.89 |
| Higher | 5,061 | 19.5 |
| Highest | 4,255 | 16.4 |
| Physical activity |  |  |
| Frequent | 6,162 | 23.74 |
| Rare | 2,064 | 7.95 |
| Never | 17,726 | 68.3 |
| Tobacco consumption |  |  |
| Never consumed tobacco | 16,171 | 62.31 |
| Currently smoking | 3,396 | 13.09 |
| Currently consumed smokeless tobacco | 5,573 | 21.47 |
| Consumed both smoking and smokeless tobacco | 813 | 3.13 |
| Alcohol consumption |  |  |
| Never consumed alcohol | 20,847 | 80.33 |
| Frequently consumed but not a heavy drinker | 1,593 | 6.14 |
| Infrequently consumed but not a heavy drinker | 3,329 | 12.83 |
| Heavy drinker | 183 | 0.71 |
| Hypertension |  |  |
| No | 11,496 | 44.3 |
| Yes | 14,456 | 55.7 |
| Diabetes |  |  |
| No | 22,226 | 85.64 |
| Yes | 3,726 | 14.36 |
| Heart disease |  |  |
| No | 24,656 | 95.01 |
| Yes | 1,296 | 4.99 |
| Stroke |  |  |
| No | 25,360 | 97.72 |
| Yes | 592 | 2.28 |
| SRH |  |  |
| Good | 19,914 | 76.74 |
| Poor | 6,038 | 23.26 |
| Region |  |  |
| North | 3,326 | 12.81 |
| West | 4,345 | 16.74 |
| Northeast | 722 | 2.78 |
| East | 6,262 | 24.13 |
| Central | 5,604 | 21.59 |
| South | 5,694 | 21.94 |

**Prevalence of excess weight** **among older adults based on socio-demographic and non-communicable diseases**

**Table 3** shows the prevalence of excess weight among older adults based on socioeconomic, behavioural and health characteristics. The prevalence of excess weight was higher among female (27%) than in male (18%) older adults in India. Respondents living in urban areas had a higher prevalence of excess weight than rural areas (40% vs. 16%). Prevalence of excess weight was high among other religions (32%) and other castes (29%), respectively. A higher percentage of older adults who had higher education (42%) and were currently in a marital union (24%) had excess weight. The prevalence of excess weight was high among older adults belonging to the richest quintile (33%). Surprisingly, 28% and 25% of the older adults who never consumed any tobacco or alcohol had excess weight. A higher percentage of older adults with hypertension (30%), diabetes (46%), and heart diseases (41%) had excess weight. Additionally, the prevalence of excess weight was higher among the respondents who had a stroke (26) and never did physical activity (24%). Again, a slightly higher percentage of older adults who reported good SRH had excess weight (24%) compared to those who reported poor SRH (20%). The prevalence of excess weight was the highest in the south region (34%), followed by the west (29%) and north region (27%).

| Table 3 Prevalence of excess weight among older adults (aged 60 years and above) based on socioeconomic, behavioral and health characteristics, India 2017-18 | | | | |
| --- | --- | --- | --- | --- |
| Socio-demographic characteristics |  |  | |  |
|  | **Prevalence (%)** | **95 % CI** | | ***p*-value** |
|  |  | **Lower** | **Upper** |  |
| Sex |  |  |  | < 0.001 |
| Male | 17.58 | 16.32 | 18.91 |  |
| Female | 26.91 | 24.75 | 29.18 |  |
| Place of residence |  |  |  | < 0.001 |
| Rural | 15.71 | 14.91 | 16.55 |  |
| Urban | 40.03 | 36.49 | 43.68 |  |
| Religion |  |  |  | < 0.001 |
| Hindu | 22.42 | 20.82 | 24.11 |  |
| Muslim | 21.91 | 19.55 | 24.46 |  |
| Christian | 22.95 | 19.31 | 27.06 |  |
| Others | 31.72 | 28.07 | 35.6 |  |
| Caste |  |  |  | < 0.001 |
| Schedule caste | 15.56 | 13.67 | 17.65 |  |
| Schedule tribe | 8.26 | 6.85 | 9.91 |  |
| Other backward class | 23.96 | 21.35 | 26.79 |  |
| Others | 29.43 | 27.84 | 31.07 |  |
| Education |  |  |  | < 0.001 |
| No education | 17.1 | 16.13 | 18.11 |  |
| Up to primary | 26.29 | 23.54 | 29.24 |  |
| Up to secondary | 35.46 | 26.15 | 46.03 |  |
| Secondary & above | 41.67 | 36.78 | 46.73 |  |
| Marital status |  |  |  | 0.001 |
| Currently in union | 23.19 | 21.99 | 24.44 |  |
| Currently not in union | 21.95 | 19.02 | 25.21 |  |
| Wealth |  |  |  | < 0.001 |
| Poorest | 14.59 | 13.15 | 16.17 |  |
| Poorer | 18.44 | 16.77 | 20.24 |  |
| Middle | 22.57 | 20.16 | 25.17 |  |
| Richer | 28.15 | 24.15 | 32.53 |  |
| Richest | 32.79 | 28.47 | 37.43 |  |
| Physical activity |  |  |  | < 0.001 |
| Frequent | 20.46 | 17.92 | 23.25 |  |
| Rare | 18.11 | 15.75 | 20.74 |  |
| Never | 24.04 | 22.3 | 25.87 |  |
| Tobacco consumption |  |  |  | < 0.001 |
| Never consumed tobacco | 28.19 | 26.21 | 30.25 |  |
| Currently smoking | 9.73 | 8.3 | 11.36 |  |
| Currently consumed smokeless tobacco | 16.51 | 14.84 | 18.34 |  |
| Consumed both smoking and smokeless tobacco | 10.85 | 8.52 | 13.72 |  |
| Alcohol consumption |  |  |  | < 0.001 |
| Never consumed alcohol | 25.32 | 23.69 | 27.02 |  |
| Frequently consumed but not a heavy drinker | 17.51 | 15.05 | 20.27 |  |
| Infrequently consumed but not a heavy drinker | 9.83 | 8.38 | 11.49 |  |
| Heavy drinker | 6.72 | 3.89 | 11.35 |  |
| Hypertension |  |  |  | < 0.001 |
| No | 13.09 | 11.93 | 14.33 |  |
| Yes | 30.38 | 28.27 | 32.58 |  |
| Diabetes |  |  |  | < 0.001 |
| No | 18.75 | 17.83 | 19.72 |  |
| Yes | 46.38 | 40.69 | 52.16 |  |
| Heart disease |  |  |  | < 0.001 |
| No | 21.74 | 20.52 | 23.01 |  |
| Yes | 41.35 | 30.17 | 53.5 |  |
| Stroke |  |  |  | 0.002 |
| No | 22.64 | 21.24 | 24.1 |  |
| Yes | 26.25 | 21.51 | 31.62 |  |
| SRH |  |  |  | 0.034 |
| Good | 23.5 | 21.79 | 25.3 |  |
| Poor | 20.15 | 18.74 | 21.63 |  |
| Region |  |  |  | < 0.001 |
| North | 27.4 | 25.78 | 29.09 |  |
| West | 28.91 | 26.61 | 31.34 |  |
| Northeast | 14.44 | 12.62 | 16.48 |  |
| East | 14.23 | 12.94 | 15.62 |  |
| Central | 14.76 | 13.17 | 16.52 |  |
| South | 33.47 | 28.75 | 38.56 |  |

***Note****. p-value based on Pearson Chi-square (χ²) tests.*

**State-wise prevalence of excess weight** **among older adults in India**

**Figure 2** provides the state-wise spatial distribution of the prevalence of excess weight in India. The states with the highest prevalence of excess weight, where at least 33% of people had excess weight, were Kerala, Karnataka, Himachal Pradesh, Punjab, and Sikkim. A high percentage (19% - 33%) of older people with excess weight belonged to the states like Tamil Nadu, Andhra Pradesh, Telangana, Maharashtra, Gujrat, Haryana, Jammu and Kashmir, and Manipur. A relatively lower prevalence of excess weight (15%-19%) among older adults was found in the states like Rajasthan, Odisha, Arunachala Pradesh, Uttarakhand, and Nagaland. The prevalence of excess weight was further lower (1%-15%) among the older adults in states including Bihar, Jharkhand, Madhya Pradesh, West Bengal, and Uttar Pradesh.

**Fig 2 Prevalence of excess weight** **among older adults in India by its states using LASI Wave 1 data**

**Factors associated with excess weight among older adults in India**

**Table 4** depicts the results obtained from the logistic regression analysis of the socio-demographic, behavioural and health-related factors associated with excess weight among older adults in India. Model-1 presents unadjusted estimates whereas, model 2 provides the adjusted estimates.

After adjusting for the selected covariates, the odds of excess weight were significantly higher among older females than males [OR: 2.17, 95% CI: 1.86, 2.54]. Similarly, the likelihood of excess weight was 1.93 times higher among the older adults who were living in urban areas with reference to their rural counterparts [OR: 1.93; 95% CI: 1.69, 2.21]. Older adults who belonged to Hindu and Muslim religious affiliations had a 0.77 [OR: 0.77, 95% CI: 0.62, 0.95] and 0.64 times [OR: 0.64, 95% CI: 0.44, 0.93] lower likelihood of excess weight compared to older adults of other religions. Compared to the other castes, older adults who belonged to the scheduled castes [OR: 0.75, 95% CI: 0.62, 0.91], scheduled tribes [OR: 0.40, 95% CI: 0.32, 0.51], and other backward class [OR: 0.84, 95% CI: 0.74, 0.97] had a significantly lower likelihood of excess weight. The increasing level of education had a significant positive association with the likelihood of excess weight. Similarly, Older adults belonged to the richest stratum [OR: 1.77, 95% CI: 1.46, 2.15], richer stratum [OR: 1.72, 95% CI: 1.41, 2.10], middle stratum [OR: 1.45, 95% CI: 1.20, 1.76], and poorer stratum [OR: 1.29, 95% CI: 1.06, 1.56] of household wealth index had a significantly higher likelihood of excess weight than older adults of poorest stratum. Older adults who had noncommunicable chronic diseases like hypertension, diabetes and heart diseases were 2.02 times [OR: 2.02, 95% CI: 1.78, 2.30], 1.87 times [OR: 1.87, 95% CI: 1.60, 2.18] and 1.38 times [OR: 1.38, 95% CI: 1.07, 1.78] higher likely to have excess weight than their counterparts who did not have any noncommunicable disease. Contrarily, older adults who had a poor SRH had lower odds of excess weight [OR: 0.78, 95% CI: 0.68,0.89] compared to those who had a good SRH. Also, people who consumed both smoking and smokeless tobacco were less likely to have excess weight than people who never consumed tobacco [OR: 0.69, 95% CI: 0.50, 0.96]. Compared to the northern region, older adults living in the North-eastern [OR: 0.54, 95% CI: 0.44, 0.67], eastern [OR: 0.52, 95% CI: 0.44, 0.61] and central region [OR: 0.60, 95% CI: 0.50, 0.72] had lower odds of excess weight.

| Table 4 Factors associated with excess weight among older adults (60 years and above) in India using logistic regression models, LASI, 2017-18 | | | | | | |
| --- | --- | --- | --- | --- | --- | --- |
| Predictors | **b** | **Model - 1** | **p- value** | **b** | **Model - 2** | **p- value** |
|  |  | **Crude OR (95% CI)** |  |  | **Adjusted OR (95% CI)** |  |
| Sex |  |  |  |  |  |  |
| Male | ref |  |  | ref |  |  |
| Female | 0.54 | 1.72 [1.49, 1.99] | < 0.001 | 0.78 | 2.17 [1.86, 2.54] | < 0.001 |
| Place of residence |  |  |  |  |  |  |
| Rural | ref |  |  | ref |  |  |
| Urban | 1.27 | 3.58 [3.04, 4.21] | < 0.001 | 0.66 | 1.93 [1.69, 2.21] | < 0.001 |
| Religion |  |  |  |  |  |  |
| Hindu | -0.47 | 0.62 [0.51, 0.76] | < 0.001 | -0.26 | 0.77 [0.62, 0.95] | < 0.05 |
| Muslim | -0.5 | 0.60 [ 0.48, 0.76] | < 0.001 | -0.2 | 0.82 [0.62, 1.07] | 0.148 |
| Christian | -0.44 | 0.64 [0.49, 0.85] | < 0.005 | -0.45 | 0.64 [0.44, 0.93] | < 0.05 |
| Others | ref |  |  | ref |  |  |
| Caste |  |  |  |  |  |  |
| Schedule caste | -0.82 | 0.44 [0.37, 0.52] | < 0.001 | -0.28 | 0.75 [0.62, 0.91] | < 0.005 |
| Schedule tribe | -1.53 | 0.22 [0.17, 0.27] | < 0.001 | -0.91 | 0.40 [0.32, 0.51] | < 0.001 |
| Other backward class | -0.28 | 0.76 [0.64, 0.89] | 0.001 | -0.17 | 0.84 [0.74, 0.97] | < 0.05 |
| Others | ref |  |  | ref |  |  |
| Education |  |  |  |  |  |  |
| No education | ref |  |  | ref |  |  |
| Up to primary | 0.55 | 1.73 [1.47, 2.04] | < 0.001 | 0.35 | 1.42 [1.19, 1.69] | < 0.001 |
| Up to secondary | 0.98 | 2.66 [1.71, 4.16] | < 0.001 | 0.84 | 2.33 [1.77, 3.06] | < 0.001 |
| Secondary & above | 1.24 | 3.46 [2.79, 4.30] | < 0.001 | 0.82 | 2.27 [1.87, 2.76] | < 0.001 |
| Marital status |  |  |  |  |  |  |
| Currently in union | ref |  |  |  |  |  |
| Currently not in union | -0.07 | 0.93 [0.77, 1.13] | 0.471 | -0.4 | 0.67 [0.59, 0.76] | < 0.001 |
| Wealth Index |  |  |  |  |  |  |
| Poorest | ref |  |  | ref |  |  |
| Poorer | 0.28 | 1.32 [1.12,1.56] | 0.001 | 0.25 | 1.29 [1.06, 1.56] | < 0.05 |
| Middle | 0.53 | 1.71 [1.41, 2.06] | < 0.001 | 0.37 | 1.45 [1.20, 1.76] | < 0.001 |
| Richer | 0.83 | 2.29 [1.80, 2.92] | < 0.001 | 0.54 | 1.72 [1.41, 2.10] | < 0.001 |
| Richest | 1.05 | 2.86 [2.25, 3.62] | < 0.001 | 0.57 | 1.77 [1.46, 2.15] | < 0.001 |
| Physical activity |  |  |  |  |  |  |
| Frequent | ref |  |  | ref |  |  |
| Rare | -0.15 | 0.86 [0.68, 1.09] | 0.207 | -0.74 | 0.93 [0.70, 1.23] | 0.603 |
| Never | 0.21 | 1.23 [1.02, 1.49] | < 0.005 | 0.003 | 1.00 [0.86,1.17] | 0.971 |
| Tobacco consumption |  |  |  |  |  |  |
| Never consumed tobacco | ref |  |  | ref |  |  |
| Currently smoking | -1.29 | 0.27 [0.22, 0.34] | < 0.001 | -0.86 | 0.42 [0.11, 1.62] | 0.21 |
| Currently consumed smokeless tobacco | -0.66 | 0.50 [0.43, 0.59] | < 0.001 | -0.12 | 0.88 [0.75, 1.03] | 0.123 |
| Consumed both smoking and smokeless tobacco | -1.17 | 0.31 [0.23, 0.41] | < 0.001 | -0.37 | 0.69 [0.50, 0.96] | < 0.05 |
| Alcohol consumption |  |  |  |  |  |  |
| Never consumed alcohol | ref |  |  | ref |  |  |
| Frequently consumed but not a heavy drinker | -0.47 | 0.63 [0.51, 0.77] | < 0.001 | 0.13 | 1.14 [0.88, 1.47] | 0.325 |
| Infrequently consumed but not a heavy drinker | -1.14 | 0.32 [0.26, 0.39] | < 0.001 | 0.23 | 1.26 [0.33, 4.90] | 0.736 |
| Heavy drinker | -1.55 | 0.21 [0.12, 0.38] | < 0.001 | -0.55 | 0.58 [0.30, 1.11] | 0.099 |
| Hypertension |  |  |  |  |  |  |
| No | ref |  |  | ref |  |  |
| Yes | 1.06 | 2.90 [2.50, 3.36] | < 0.001 | 0.7 | 2.02 [1.78, 2.30] | < 0.001 |
| Diabetes |  |  |  |  |  |  |
| No | ref |  |  | ref |  |  |
| Yes | 1.32 | 3.74 [2.95, 4.76] | < 0.001 | 0.63 | 1.87 [1.60, 2.18] | < 0.001 |
| Heart disease |  |  |  |  |  |  |
| No | ref |  |  | ref |  |  |
| Yes | 0.93 | 2.53 [1.55, 4.16] | < 0.001 | 0.32 | 1.38 [1.07, 1.78] | < 0.05 |
| Stroke |  |  |  |  |  |  |
| No | ref |  |  | ref |  |  |
| Yes | 0.2 | 1.21 [0.92, 1.60] | 0.161 | 0.02 | 1.02 [0.76, 1.38] | 0.878 |
| ^a^ SRH |  |  |  |  |  |  |
| Good | ref |  |  | ref |  |  |
| Poor | -0.2 | 0.82 [0.72, 0.94] | < 0.005 | -0.25 | 0.78 [0.68,0.89] | < 0.001 |
| Region |  |  |  |  |  |  |
| North | ref |  |  | ref |  |  |
| West | 0.07 | 1.08 [0.93 - 1.24] | 0.303 | -0.11 | 0.89 [0.76,1.05] | 0.181 |
| Northeast | -0.8 | 0.45 [0.37 - 0.53] | < 0.001 | -0.62 | 0.54 [0.44,0.67] | < 0.001 |
| East | -0.82 | 0.44 [0.38 - 0.50] | < 0.001 | -0.65 | 0.52 [0.44,0.61] | < 0.001 |
| Central | -0.8 | 0.46 [0.39 - 0.54] | < 0.001 | -0.51 | 0.60 [0.50,0.72] | < 0.001 |
| South | 0.29 | 1.33 [1.05 - 1.69] | <0.05 | 0.13 | 1.14 [0.95,1.36] | 0.164 |

********OR: Odd Ratio; CI: Confidence Interval; ref: reference; b: coefficient; ^a^ SRH: Self-rated health*

**Discussion**

This study is an attempt to assess the prevalence of excess weight and to examine the socio-demographic, behavioral and health-related factors associated with excess weight among older Indian adults. While India ranked 107th among 121 countries on the 2022 Global Hunger Index (GHI), the current findings indicated that one in every four Indians aged 60 years and above had excess weight. Such a higher prevalence of excess weight among older adults is intriguing and motivates us to dig more into this significant public health problem of overweight/obesity. Further, we observed that the percentage of women with overweight/obesity is significantly higher than that of males [43-45]. In developing countries, women tend to be less active than men, which can contribute to their increased risk of being overweight and obese; however, in high-income countries, females are not disadvantaged when it comes to physical inactivity and healthy food habits; as resources and opportunities for men and women have become increasingly alike over the years [46].

In India, the prevalence of excess weight is significantly related to their social and economic standing. Populations from a higher caste, households with the richest wealth quintile, higher level of education, and residing in urban areas have a greater prevalence of excess weight than that belonging to lower castes, lower economic stratum, rural areas and are less educated [47, 48]. Indians from higher socioeconomic strata consume more calories and fat in their diets and exercise less than those from lower socioeconomic levels, which leads to a higher prevalence of excess weight [49-51]. All of these variables are interconnected, and in the Indian context, upper-caste individuals are often recognised to have better levels of education and economic prosperity than lower-caste individuals. Urban individuals also exhibit comparable traits. These people frequently consume a lot of calories and put forth little effort, which may cause overweight/obesity. The majority of India's lower caste (scheduled tribe) and less wealthy population engages in physical exercise since their economy is based mostly on agriculture. Most of the earlier studies on overweight and obesity in India also portray similar findings [45,52,53].

As previously indicated, this study found that as education levels rise, the likelihood of having excess weight also rises significantly. Many studies support this finding, but few have attempted to determine if education has any beneficial effects on obesity [48]. Siddiqui et al. (2016) [52] found that there is a negative correlation between years of education and the likelihood of having excess weight above a certain threshold level of educational attainment. They found that the likelihood of BMI initially increases with an increasing level of education up to a certain point and then starts declining gradually. This is brought on by a rise in health concerns and awareness among highly educated people [52].

This study further demonstrates that there is a strong correlation between excess weight and chronic diseases, such as hypertension, diabetes, and heart disease. Obesity significantly affects all three ailments, including hypertension [53-58], type 2 diabetes mellitus [53, 54, 59-61], and heart disease [62-64], all common chronic diseases that are highly costly to our society in terms of health care expenditures and premature morbidity and mortality [65]. It is highly typical for overweight and obese people also to have these chronic conditions. Numerous research has looked at the relationship between obesity and the illnesses mentioned above individually and has come to similar conclusions [66-68].

Physical activity and excess weight have been proven to have a negative correlation in the unadjusted model; those who are constantly active have a lesser risk of having excess weight, and comparable results have been reported in other investigations [69-72]. Enigmatically, we discovered that there was no such notable difference in the impact of physical activity on excess weight when we controlled various socioeconomic, demographic, and other personal factors, including numerous chronic conditions. Analysis in this study demonstrates that older women are more likely to have excess weight compared to men and that most women engage in less physical activity than men. This is one of the reasons that the association of physical activity with excess weight deteriorates when sex and other factors are controlled. In order to support this assertion, we used a different model without the sex variable. Surprisingly, the results were in line with our expectations, and we discovered that physical activity is a negative predictor of excess weight. Since the focus of this study was not specifically on the relationship between physical activity and excess weight, we did not provide the model individually in the paper. However, the model's results are available in **Appendix 1**.

In this study, smoking behaviour was found to be negatively associated with excess weight. In general, smoking is believed to be a risk factor for weight loss, and many studies on smoking behaviour and body weight revealed that smoking behaviour reduces body weight as smoking is associated with greater energy expenditure, suppressed appetite, and several morbid conditions [73,74].

According to the study, people with excess weight are more likely to report having poorer health, and this link holds even after adjusting for the impact of other relevant characteristics such as demographics, socioeconomic position, chronic diseases, and lifestyle choices. The findings of other research across the globe are likewise consistent [75,76]. According to studies, a socioeconomic gradient in health manifests in a way that persons in the lower social strata have worse health [77]; one such illness that disproportionately affects those from lower socioeconomic backgrounds is higher BMI and obesity [78-82]. Given the association between excess weight and self-rated health, it is possible that other underlying characteristics like socioeconomic status have an impact on self-reported health through their influence on excess weight.

A key strength of this research is that we included various socioeconomic, behavioral and health-related correlates that play an important role as significant determinants of excess weight among older Indians, irrespective of various regions. Another important strength of our study is the inclusion of a nationally representative sample of community-dwelling older adults in India. However, this study has some limitations too. First, the cross-sectional design of the study restricts our ability to infer the causality in the observed associations, and the self-report nature of many of the correlates may lead to reporting biases. Second, several factors such as dietary patterns, food habits, food preferences and food security were not considered in our study. Future research should consider these aspects while analysing the factors associated with excess weight among older adults. Third, BMI measurement in our study does not differentiate between lean or fat mass which can have distinct clinical and biological significance. Fourth, body fat in younger, middle-aged and older adults can have different implications and thus, future studies should focus on age-stratified analysis of factors associated with excess weight. Finally, people from Asian countries have more body fat than people from other regions of the world, and the higher prevalence of excess weight might partially be attributed to the standard cut-off we used. Further investigation is required using the Asian-specific BMI classification and multiple categories of BMI including underweight.

**Conclusion**

Findings suggest that females, urban place of residence, higher levels of education, higher household economic status, and being diagnosed with hypertension, diabetes and heart disease were associated with higher prevalence of excess weight among older adults. As such, the difficulties of implementing programs and policies that would lessen the negative consequences of morbidity associated with excess weight among the older population must be addressed. The findings further highlight that additional healthy lifestyle practices are needed for the prevention and reduction of excess weight among older adults who have comorbid conditions, such as diabetes and hypertension. In order to improve older adults' functional status and prevent them from becoming disabled and consequently experiencing poor quality of life, policymakers and healthcare professionals must consider interventions addressing excess weight while developing disease-specific management programmes.

**Declarations**

**Funding**

The present research did not receive any grant from any funding agency, commercial entity, or not-for-profit organization.

**Competing interests**

All authors declare no potential competing interests.

**Data availability**

The data are available in the public domain and freely accessible from the Gateway to Global Aging Data ([www.g2aging.org](http://www.g2aging.org)). The data are also available in the International Institute for Population Sciences (IIPS) data repository of Longitudinal Aging Study in India, upon request to IIPS, Mumbai, <https://www.iipsindia.ac.in/content/LASI-data>.

**Author Contributions**

**Conceptualization:** Amiya Saha

**Data curation:** Amiya Saha

**Formal analysis:** Amiya Saha,

**Methodology:** Amiya Saha, Bittu Mandal, Papai Barman

**Software:** Amiya Saha

**Supervision:** Amiya Saha, Bittu Mandal, Papai Barman and Mihir Adhikary

**Writing – original draft:** Amiya Saha, T. Muhammad, Bittu Mandal, Mihir Adhikary and Papai Barman

**Writing –** **review & editing**: Amiya Saha, T. Muhammad and Papai Barman

**Consent for publication**

Not applicable

**Acknowledgements**

None

**References**

1. Lutz W, Sanderson W, Scherbov S. The coming acceleration of global population ageing. Nature. 2008 Feb 7;451(7179):716-9. doi: 10.1038/nature06516. Epub 2008 Jan 20. PMID: 18204438.
2. United Nations, “World Population Prospects - Population Division - United Nations,” *World Population Prospects - 2015 Revision*, pp. 1–5, 2015, Available: <https://www.ptonline.com/articles/how-to-get-better-mfi-results>
3. Balachandran A, de Beer J, James KS, van Wissen L, Janssen F. Comparison of Population Aging in Europe and Asia Using a Time-Consistent and Comparative Aging Measure. J Aging Health. 2020 Jun/Jul;32(5-6):340-351. doi: 10.1177/0898264318824180. Epub 2019 Jan 17. PMID: 30651037; PMCID: PMC7322980.
4. United Nations Population Division. The 2015 Revision of the UN’s World Population Projections. Popul. Dev. Rev. 2015; 41:557-61.
5. Stuckler D. Population causes and consequences of leading chronic diseases: a comparative analysis of prevailing explanations. Milbank Q. 2008 Jun;86(2):273-326. doi: 10.1111/j.1468-0009.2008.00522. x. PMID: 18522614; PMCID: PMC2690359.
6. Janssen F, Trias-Llimós S, Kunst AE. The combined impact of smoking, obesity and alcohol on life-expectancy trends in Europe. Int J Epidemiol. 2021 Jul 9;50(3):931-941. doi: 10.1093/ije/dyaa273. PMID: 33432332; PMCID: PMC8271206.
7. Karnik S, Kanekar A. Childhood obesity: a global public health crisis. Int J Prev Med. 2012 Jan;3(1):1-7. PMID: 22506094; PMCID: PMC3278864.
8. Arroyo-Johnson C, Mincey KD. Obesity Epidemiology Worldwide. Gastroenterol Clin North Am. 2016 Dec;45(4):571-579. doi: 10.1016/j.gtc.2016.07.012. PMID: 27837773; PMCID: PMC5599163.
9. Amarya S, Singh K, Sabharwal M. Health consequences of obesity in the elderly. Journal of Clinical Gerontology and Geriatrics. 2014 Sep 1;5(3):63-7.
10. Lomangino K. Obesity and Mortality: Seven Explanations For a Controversial Meta-Analysis. Clinical Nutrition Insight. 2013 Apr 1;39(4):6-7.
11. Hruby A, Hu FB. The Epidemiology of Obesity: A Big Picture. Pharmacoeconomics. 2015 Jul;33(7):673-89. doi: 10.1007/s40273-014-0243-x. PMID: 25471927; PMCID: PMC4859313.
12. Bhurosy T, Jeewon R. Overweight and obesity epidemic in developing countries: a problem with diet, physical activity, or socioeconomic status? ScientificWorldJournal. 2014;2014:964236. doi: 10.1155/2014/964236. Epub 2014 Oct 14. PMID: 25379554; PMCID: PMC4212551.
13. DeJesus RS, Croghan IT, Jacobson DJ, Fan C, St Sauver J. Incidence of Obesity at 1 and 3 Years Among Community Dwelling Adults: A Population-Based Study. J Prim Care Community Health. 2022 Jan-Dec;13:21501319211068632. doi: 10.1177/21501319211068632. PMID: 34986686; PMCID: PMC8744193.
14. Prentice AM, Jebb SA. Obesity in Britain: gluttony or sloth? BMJ. 1995 Aug 12;311(7002):437-9. doi: 10.1136/bmj.311.7002.437. PMID: 7640595; PMCID: PMC2550498.
15. Dai H, Alsalhe TA, Chalghaf N, Riccò M, Bragazzi NL, Wu J. The global burden of disease attributable to high body mass index in 195 countries and territories, 1990-2017: An analysis of the Global Burden of Disease Study. PLoS Med. 2020 Jul 28;17(7):e1003198. doi: 10.1371/journal.pmed.1003198. PMID: 32722671; PMCID: PMC7386577.
16. GBD 2015 Obesity Collaborators. Health effects of overweight and obesity in 195 countries over 25 years. New England journal of medicine. 2017 Jul 6;377(1):13-27.
17. Ahirwar R, Mondal PR. Prevalence of obesity in India: A systematic review. Diabetes Metab Syndr. 2019 Jan-Feb;13(1):318-321. doi: 10.1016/j.dsx.2018.08.032. Epub 2018 Sep 21. PMID: 30641719.
18. Samal S, Panigrahi P, Dutta A. Social epidemiology of excess weight and central adiposity in older Indians: analysis of Study on global AGEing and adult health (SAGE). BMJ Open. 2015 Nov 26;5(11):e008608. doi: 10.1136/bmjopen-2015-008608. PMID: 26610757; PMCID: PMC4679837.
19. Chapman IM. Obesity in old age. Front Horm Res. 2008;36:97-106. doi: 10.1159/000115358. PMID: 18230897.
20. Genton L, Karsegard VL, Chevalley T, Kossovsky MP, Darmon P, Pichard C. Body composition changes over 9 years in healthy elderly subjects and impact of physical activity. Clin Nutr. 2011 Aug;30(4):436-42. doi: 10.1016/j.clnu.2011.01.009. Epub 2011 Feb 15. PMID: 21324569.
21. Rajkamal R, Seralathan M, Jayakiruthiga S. Prevalence and factors associated with overweight and obesity among elderly people in a semi‐urban area of Chennai. Int J Community Med Public Health. 2018 Sep;5(9):3887-91.
22. Hajek A, Lehnert T, Ernst A, Lange C, Wiese B, Prokein J, Weyerer S, Werle J, Pentzek M, Fuchs A, Luck T, Bickel H, Mösch E, Heser K, Wagner M, Maier W, Scherer M, Riedel-Heller SG, König HH; AgeCoDe Study Group. Prevalence and determinants of overweight and obesity in old age in Germany. BMC Geriatr. 2015 Jul 14;15:83. doi: 10.1186/s12877-015-0081-5. PMID: 26170016; PMCID: PMC4499904.
23. Chernoff R. Nutrition and health promotion in older adults. J Gerontol A Biol Sci Med Sci. 2001 Oct;56 Spec No 2:47-53. doi: 10.1093/gerona/56.suppl_2.47. PMID: 11730237.
24. Visscher TL, Seidell JC. The public health impact of obesity. Annu Rev Public Health. 2001;22:355-75. doi: 10.1146/annurev.publhealth.22.1.355. PMID: 11274526.
25. Visscher TL, Seidell JC. The public health impact of obesity. Annu Rev Public Health. 2001;22:355-75. doi: 10.1146/annurev.publhealth.22.1.355. PMID: 11274526.
26. Gillis KJ, Hirdes JP. The quality of life implications of health practices among older adults: evidence from the 1991 Canadian General Social Survey. Canadian Journal on Aging/La Revue canadienne du vieillissement. 1996 Jan;15(2):299-314.
27. Jensen GL, Rogers J. Obesity in older persons. J Am Diet Assoc. 1998 Nov;98(11):1308-11. doi: 10.1016/S0002-8223(98)00293-4. PMID: 9813588.
28. Himes CL. Obesity, disease, and functional limitation in later life. Demography. 2000 Feb;37(1):73-82. PMID: 10748990.
29. Flegal KM, Carroll MD, Ogden CL, Johnson CL. Prevalence and trends in obesity among US adults, 1999-2000. JAMA. 2002 Oct 9;288(14):1723-7. doi: 10.1001/jama.288.14.1723. PMID: 12365955.
30. Hajek A, König HH. Are changes in body-mass-index associated with changes in depressive symptoms? Findings of a population-based longitudinal study among older Germans. BMC Psychiatry. 2018 Jun 8;18(1):182. doi: 10.1186/s12888-018-1748-1. PMID: 29884148; PMCID: PMC5994125.
31. Spahlholz J, Baer N, König HH, Riedel-Heller SG, Luck-Sikorski C. Obesity and discrimination - a systematic review and meta-analysis of observational studies. Obes Rev. 2016 Jan;17(1):43-55. doi: 10.1111/obr.12343. Epub 2015 Nov 24. PMID: 26596238.
32. Hajek A, König HH. Obesity and loneliness. Findings from a longitudinal population-based study in the second half of life in Germany. Psychogeriatrics. 2019 Mar;19(2):135-140. doi: 10.1111/psyg.12375. Epub 2018 Oct 8. PMID: 30294939.
33. König HH, Lehnert T, Brenner H, Schöttker B, Quinzler R, Haefeli WE, Matschinger H, Heider D. Health service use and costs associated with excess weight in older adults in Germany. Age Ageing. 2015 Jul;44(4):616-23. doi: 10.1093/ageing/afu120. Epub 2015 Mar 31. PMID: 25829392.
34. Rajkamal R, Singh Z, Stalin P, Muthurajesh E. Prevalence and determinants of overweight and obesity among elderly population in an urban area of Puducherry. Int J Med Sci Public Health. 2015 Mar 1;4(3):369-72.
35. International Institute for Population Sciences (IIPS). National Programme for Health Care of Elderly (NPHCE), MoHFW, Harvard T. H. Chan School of Public Health (HSPH) and the University of Southern California (USC). 2020;1–632.
36. Wen CP, David Cheng TY, Tsai SP, Chan HT, Hsu HL, Hsu CC, Eriksen MP. Are Asians at greater mortality risks for being overweight than Caucasians? Redefining obesity for Asians. Public Health Nutr. 2009 Apr;12(4):497-506. doi: 10.1017/S1368980008002802. Epub 2008 Jun 12. PMID: 18547457.
37. Banerjee S, Kumar P, Srivastava S, Banerjee A. Association of anthropometric measures of obesity and physical activity with cardio-vascular diseases among older adults: Evidence from a cross-sectional survey, 2017-18. PLoS One. 2021 Dec 15;16(12):e0260148. doi: 10.1371/journal.pone.0260148. PMID: 34910748; PMCID: PMC8673620.
38. Rahaman M, Rana MJ, Roy A, Chouhan P. Spatial heterogeneity and socio-economic correlates of unmet need for spacing contraception in India: Evidences from National Family Health Survey, 2015-16. Clinical Epidemiology and Global Health. 2022 May 1;15:101012.
39. Srivastava S, Singh SK, Kumar M, Muhammad T. Distinguishing between household headship with and without power and its association with subjective well-being among older adults: an analytical cross-sectional study in India. BMC Geriatr. 2021 May 12;21(1):304. doi: 10.1186/s12877-021-02256-0. PMID: 33980164; PMCID: PMC8114520.
40. Srivastava S, Kumar S. Does socio-economic inequality exist in micro-nutrients supplementation among children aged 6-59 months in India? Evidence from National Family Health Survey 2005-06 and 2015-16. BMC Public Health. 2021 Mar 19;21(1):545. doi: 10.1186/s12889-021-10601-6. PMID: 33740942; PMCID: PMC7980608.
41. Saha A, Rahaman M, Mandal B, Biswas S, Govil D. Rural urban differences in self-rated health among older adults: examining the role of marital status and living arrangements. BMC Public Health. 2022 Nov 25;22(1):2175. doi: 10.1186/s12889-022-14569-9. PMID: 36434537; PMCID: PMC9700952.
42. Chobanian AV, Bakris GL, Black HR, Cushman WC, Green LA, Izzo JL Jr, Jones DW, Materson BJ, Oparil S, Wright JT Jr, Roccella EJ; National Heart, Lung, and Blood Institute Joint National Committee on Prevention, Detection, Evaluation, and Treatment of High Blood Pressure; National High Blood Pressure Education Program Coordinating Committee. The Seventh Report of the Joint National Committee on Prevention, Detection, Evaluation, and Treatment of High Blood Pressure: the JNC 7 report. JAMA. 2003 May 21;289(19):2560-72. doi: 10.1001/jama.289.19.2560. Epub 2003 May 14. Erratum in: JAMA. 2003 Jul 9;290(2):197. PMID: 12748199.
43. Ahirwar R, Mondal PR. Prevalence of obesity in India: A systematic review. Diabetes Metab Syndr. 2019 Jan-Feb;13(1):318-321. doi: 10.1016/j.dsx.2018.08.032. Epub 2018 Sep 21. PMID: 30641719.
44. Cooper AJ, Gupta SR, Moustafa AF, Chao AM. Sex/Gender Differences in Obesity Prevalence, Comorbidities, and Treatment. Curr Obes Rep. 2021 Dec;10(4):458-466. doi: 10.1007/s13679-021-00453-x. Epub 2021 Oct 2. PMID: 34599745.
45. Luhar S, Timaeus IM, Jones R, Cunningham S, Patel SA, Kinra S, Clarke L, Houben R. Forecasting the prevalence of overweight and obesity in India to 2040. PLoS One. 2020 Feb 24;15(2): e0229438. doi: 10.1371/journal.pone.0229438. PMID: 32092114; PMCID: PMC7039458.
46. Kanter R, Caballero B. Global gender disparities in obesity: a review. Adv Nutr. 2012 Jul 1;3(4):491-8. doi: 10.3945/an.112.002063. PMID: 22797984; PMCID: PMC3649717.
47. Dinsa GD, Goryakin Y, Fumagalli E, Suhrcke M. Obesity and socioeconomic status in developing countries: a systematic review. Obes Rev. 2012 Nov;13(11):1067-79. doi: 10.1111/j.1467-789X.2012.01017.x. Epub 2012 Jul 5. PMID: 22764734; PMCID: PMC3798095.
48. Cohen AK, Rai M, Rehkopf DH, Abrams B. Educational attainment and obesity: a systematic review. Obes Rev. 2013 Dec;14(12):989-1005. doi: 10.1111/obr.12062. Epub 2013 Jul 25. PMID: 23889851; PMCID: PMC3902051.
49. Ogden CL. Obesity and socioeconomic status in children and adolescents: United States, 2005-2008. US Department of Health and Human Services, Centers for Disease Control and Prevention, National Center for Health Statistics; 2010.
50. Misra A, Shrivastava U. Obesity and dyslipidemia in South Asians. Nutrients. 2013 Jul 16;5(7):2708-33. doi: 10.3390/nu5072708. PMID: 23863826; PMCID: PMC3738996.
51. Griffiths P, Bentley M. Women of higher socio-economic status are more likely to be overweight in Karnataka, India. Eur J Clin Nutr. 2005 Oct;59(10):1217-20. doi: 10.1038/sj.ejcn.1602228. PMID: 16077746.
52. Siddiqui MZ, Donato R. Overweight and obesity in India: policy issues from an exploratory multi-level analysis. Health Policy Plan. 2016 Jun;31(5):582-91. doi: 10.1093/heapol/czv105. Epub 2015 Nov 13. PMID: 26567124.
53. Corsi DJ, Subramanian SV. Socioeconomic Gradients and Distribution of Diabetes, Hypertension, and Obesity in India. JAMA Netw Open. 2019 Apr 5;2(4):e190411. doi: 10.1001/jamanetworkopen.2019.0411. PMID: 30951154; PMCID: PMC6450330.
54. Babu GR, Murthy GVS, Ana Y, Patel P, Deepa R, Neelon SEB, Kinra S, Reddy KS. Association of obesity with hypertension and type 2 diabetes mellitus in India: A meta-analysis of observational studies. World J Diabetes. 2018 Jan 15;9(1):40-52. doi: 10.4239/wjd.v9.i1.40. PMID: 29359028; PMCID: PMC5763039.
55. Jiang SZ, Lu W, Zong XF, Ruan HY, Liu Y. Obesity and hypertension. Exp Ther Med. 2016 Oct;12(4):2395-2399. doi: 10.3892/etm.2016.3667. Epub 2016 Sep 6. PMID: 27703502; PMCID: PMC5038894.
56. Mikhail N, Golub MS, Tuck ML. Obesity and hypertension. Prog Cardiovasc Dis. 1999 Jul-Aug;42(1):39-58. doi: 10.1016/s0033-0620(99)70008-3. PMID: 10505492.
57. Seravalle G, Grassi G. Obesity and hypertension. Pharmacol Res. 2017 Aug;122:1-7. doi: 10.1016/j.phrs.2017.05.013. Epub 2017 May 19. PMID: 28532816.
58. El-Atat F, Aneja A, Mcfarlane S, Sowers J. Obesity and hypertension. Endocrinol Metab Clin North Am. 2003 Dec;32(4):823-54. doi: 10.1016/s0889-8529(03)00070-7. PMID: 14711064.
59. Verma S, Hussain ME. Obesity and diabetes: An update. Diabetes Metab Syndr. 2017 Jan-Mar;11(1):73-79. doi: 10.1016/j.dsx.2016.06.017. Epub 2016 Jun 17. PMID: 27353549.
60. Felber JP, Golay A. Pathways from obesity to diabetes. Int J Obes Relat Metab Disord. 2002 Sep;26 Suppl 2:S39-45. doi: 10.1038/sj.ijo.0802126. PMID: 12174327.
61. Hossain P, Kawar B, El Nahas M. Obesity and diabetes in the developing world--a growing challenge. N Engl J Med. 2007 Jan 18;356(3):213-5. doi: 10.1056/NEJMp068177. Erratum in: N Engl J Med. 2007 Mar 1;356(9):973. PMID: 17229948.
62. Sowers JR. Obesity as a cardiovascular risk factor. Am J Med. 2003 Dec 8;115 Suppl 8A:37S-41S. doi: 10.1016/j.amjmed.2003.08.012. PMID: 14678864.
63. Rashid MN, Fuentes F, Touchon RC, Wehner PS. Obesity and the risk for cardiovascular disease. Prev Cardiol. 2003 Winter;6(1):42-7. doi: 10.1111/j.1520-037x.2003.01358.x. PMID: 12624562.
64. Wolf AM, Colditz GA. The cost of obesity: the US perspective. Pharmacoeconomics. 1994;5(Suppl 1):34-7. doi: 10.2165/00019053-199400051-00007. PMID: 10147247.
65. Nugent R. Chronic diseases in developing countries: health and economic burdens. Ann N Y Acad Sci. 2008;1136:70-9. doi: 10.1196/annals.1425.027. PMID: 18579877.
66. Agborsangaya CB, Ngwakongnwi E, Lahtinen M, Cooke T, Johnson JA. Multimorbidity prevalence in the general population: the role of obesity in chronic disease clustering. BMC Public Health. 2013 Dec 10;13:1161. doi: 10.1186/1471-2458-13-1161. PMID: 24325303; PMCID: PMC4029057.
67. Kearns K, Dee A, Fitzgerald AP, Doherty E, Perry IJ. Chronic disease burden associated with overweight and obesity in Ireland: the effects of a small BMI reduction at population level. BMC Public Health. 2014 Feb 10;14:143. doi: 10.1186/1471-2458-14-143. PMID: 24512151; PMCID: PMC3929131.
68. Li C, Engström G, Hedblad B, Calling S, Berglund G, Janzon L. Sex differences in the relationships between BMI, WHR and incidence of cardiovascular disease: a population-based cohort study. Int J Obes (Lond). 2006 Dec;30(12):1775-81. doi: 10.1038/sj.ijo.0803339. Epub 2006 Apr 11. PMID: 16607382.
69. Poortinga W. Perceptions of the environment, physical activity, and obesity. Soc Sci Med. 2006 Dec;63(11):2835-46. doi: 10.1016/j.socscimed.2006.07.018. Epub 2006 Sep 6. PMID: 16952415.
70. Wareham NJ, van Sluijs EM, Ekelund U. Physical activity and obesity prevention: a review of the current evidence. Proc Nutr Soc. 2005 May;64(2):229-47. doi: 10.1079/pns2005423. Erratum in: Proc Nutr Soc. 2005 Nov;64(4):581-4. PMID: 15960868.
71. Fox KR, Hillsdon M. Physical activity and obesity. Obes Rev. 2007 Mar;8 Suppl 1:115-21. doi: 10.1111/j.1467-789X.2007.00329.x. PMID: 17316313.
72. Petersen L, Schnohr P, Sørensen TI. Longitudinal study of the long-term relation between physical activity and obesity in adults. Int J Obes Relat Metab Disord. 2004 Jan;28(1):105-12. doi: 10.1038/sj.ijo.0802548. PMID: 14647181.
73. Hofstetter A, Schutz Y, Jéquier E, Wahren J. Increased 24-hour energy expenditure in cigarette smokers. N Engl J Med. 1986 Jan 9;314(2):79-82. doi: 10.1056/NEJM198601093140204. PMID: 3941694.
74. Chiolero A, Faeh D, Paccaud F, Cornuz J. Consequences of smoking for body weight, body fat distribution, and insulin resistance. Am J Clin Nutr. 2008 Apr;87(4):801-9. doi: 10.1093/ajcn/87.4.801. PMID: 18400700.
75. Prosper MH, Moczulski VL, Qureshi A. Obesity as a predictor of self-rated health. Am J Health Behav. 2009 May-Jun;33(3):319-29. doi: 10.5993/ajhb.33.3.10. PMID: 19063653.
76. Okosun IS, Choi S, Matamoros T, Dever GE. Obesity is associated with reduced self-rated general health status: evidence from a representative sample of white, black, and Hispanic Americans. Prev Med. 2001 May;32(5):429-36. doi: 10.1006/pmed.2001.0840. PMID: 11330993.
77. Starfield B. Promoting equity in health through research and understanding. Dev World Bioeth. 2004 May;4(1):76-95. doi: 10.1111/j.1471-8731.2004.00068.x. PMID: 15086375.
78. Sundquist J, Johansson SE. The influence of socioeconomic status, ethnicity and lifestyle on body mass index in a longitudinal study. Int J Epidemiol. 1998 Feb;27(1):57-63. doi: 10.1093/ije/27.1.57. PMID: 9563694.
79. Sobal J, Stunkard AJ. Socioeconomic status and obesity: a review of the literature. Psychol Bull. 1989 Mar;105(2):260-75. doi: 10.1037/0033-2909.105.2.260. PMID: 2648443.
80. Jeffery RW, French SA, Forster JL, Spry VM. Socioeconomic status differences in health behaviors related to obesity: the Healthy Worker Project. Int J Obes. 1991 Oct;15(10):689-96. PMID: 1752730.
81. Paeratakul S, Lovejoy JC, Ryan DH, Bray GA. The relation of gender, race and socioeconomic status to obesity and obesity comorbidities in a sample of US adults. Int J Obes Relat Metab Disord. 2002 Sep;26(9):1205-10. doi: 10.1038/sj.ijo.0802026. PMID: 12187397.
82. Chang VW, Lauderdale DS. Income disparities in body mass index and obesity in the United States, 1971-2002. Arch Intern Med. 2005 Oct 10;165(18):2122-8. doi: 10.1001/archinte.165.18.2122. PMID: 16217002.

**Appendix**

| Table 1 Factors associated with obesity among older adults (60 years and above) in India using logistic estimates, LASI, 2017-18 | | | | | | |
| --- | --- | --- | --- | --- | --- | --- |
| Predictors | **b** | **Model - 1** | **p- value** | **b** | **Model - 2** | **p- value** |
|  |  | **Crude OR (95% CI)** |  |  | **Adjusted OR (95% CI)** |  |
| Sex |  |  |  |  |  |  |
| Male | ref |  |  |  |  |  |
| Female | 0.54 | 1.72 [1.49, 1.99] | < 0.001 |  |  |  |
| Place of residence |  |  |  |  |  |  |
| Rural | ref |  |  | ref |  |  |
| Urban | 1.27 | 3.58 [3.04, 4.21] | < 0.001 | 0.71 | 2.03 [1.76-2.33] | < 0.001 |
| Religion |  |  |  |  |  |  |
| Hindu | -0.47 | 0.62 [0.51, 0.76] | < 0.001 | -0.22 | 0.80 [0.65-0.99] | < 0.05 |
| Muslim | -0.5 | 0.60 [ 0.48, 0.76] | < 0.001 | -0.21 | 0.81 [0.62-1.06] | 0.123 |
| Christian | -0.44 | 0.64 [0.49, 0.85] | < 0.005 | -0.36 | 0.69 [0.48-1.01] | 0.056 |
| Others | ref |  |  | Ref |  |  |
| Caste |  |  |  |  |  |  |
| Schedule caste | -0.82 | 0.44 [0.37, 0.52] | < 0.001 | -0.3 | 0.74 [0.61-0.89] | <0.005 |
| Schedule tribe | -1.53 | 0.22 [0.17, 0.27] | < 0.001 | -0.88 | 0.41 [0.33-0.53] | < 0.001 |
| Other backward class | -0.28 | 0.76 [0.64, 0.89] | 0.001 | -0.19 | 0.83 [0.72-0.95] | < 0.05 |
| Others | ref |  |  | ref |  |  |
| Education |  |  |  |  |  |  |
| No education | ref |  |  | ref |  |  |
| Up to primary | 0.55 | 1.73 [1.47, 2.04] | < 0.001 | 0.2 | 1.22 [1.02-1.45] | < 0.05 |
| Up to secondary | 0.98 | 2.66 [1.71, 4.16] | < 0.001 | 0.63 | 1.88 [1.42-2.49] | < 0.001 |
| Secondary & above | 1.24 | 3.46 [2.79, 4.30] | < 0.001 | 0.48 | 1.61 [1.35-1.92] | < 0.001 |
| Marital status |  |  |  |  |  |  |
| Currently in union | ref |  |  | ref |  |  |
| Currently not in union | -0.07 | 0.93 [0.77, 1.13] | 0.471 | -0.22 | 0.81 [0.71-0.92] | <0.005 |
| Wealth Index |  |  |  |  |  |  |
| Poorest | ref |  |  | Ref |  |  |
| Poorer | 0.28 | 1.32 [1.12,1.56] | 0.001 | 0.26 | 1.29 [1.07-1.57] | <0.005 |
| Middle | 0.53 | 1.71 [1.41, 2.06] | < 0.001 | 0.4 | 1.49 [1.23-1.81] | < 0.001 |
| Richer | 0.83 | 2.29 [1.80, 2.92] | < 0.001 | 0.57 | 1.77 [1.45-2.17] | < 0.001 |
| Richest | 1.05 | 2.86 [2.25, 3.62] | < 0.001 | 0.62 | 1.86 [1.53-2.27] | < 0.001 |
| Physical activity |  |  |  |  |  |  |
| Frequent | ref |  |  | ref |  |  |
| Rare | -0.15 | 0.86 [0.68, 1.09] | 0.207 | -0.04 | 0.96 [0.72-1.29] | 0.780 |
| Never | 0.21 | 1.23 [1.02, 1.49] | < 0.005 | 0.09 | **1.14** [1.08-1.28] | **< 0.05** |
| Tobacco consumption |  |  |  |  |  |  |
| Never consumed tobacco | ref |  |  | ref |  |  |
| Currently smoking | -1.29 | 0.27 [0.22, 0.34] | < 0.001 | -1.00 | 0.37 [0.10-1.37] | 0.135 |
| Currently consumed smokeless tobacco | -0.66 | 0.50 [0.43, 0.59] | < 0.001 | -0.25 | 0.78 [0.66-0.91] | <0.005 |
| Consumed both smoking and smokeless tobacco | -1.17 | 0.31 [0.23, 0.41] | < 0.001 | -0.67 | 0.51 [0.38-0.70] | < 0.001 |
| Alcohol consumption |  |  |  |  |  |  |
| Never consumed alcohol | ref |  |  | ref |  |  |
| Frequently consumed but not a heavy drinker | -0.47 | 0.63 [0.51, 0.77] | < 0.001 | -0.17 | 0.85 [0.65-1.10] | 0.214 |
| Infrequently consumed but not a heavy drinker | -1.14 | 0.32 [0.26, 0.39] | < 0.001 | -0.02 | 0.98 [0.26-3.71] | 0.981 |
| Heavy drinker | -1.55 | 0.21 [0.12, 0.38] | < 0.001 | -0.85 | 0.43 [0.23-0.80] | <0.005 |
| Hypertension |  |  |  |  |  |  |
| No | ref |  |  | ref |  |  |
| Yes | 1.06 | 2.90 [2.50, 3.36] | < 0.001 | 0.73 | 2.07 [1.82-2.35] | < 0.001 |
| Diabetes |  |  |  |  |  |  |
| No | ref |  |  | ref |  |  |
| Yes | 1.32 | 3.74 [2.95, 4.76] | < 0.001 | 0.61 | 1.84 [1.57-2.16] | < 0.001 |
| Heart disease |  |  |  |  |  |  |
| No | ref |  |  | ref |  |  |
| Yes | 0.93 | 2.53 [1.55, 4.16] | < 0.001 | 0.32 | 1.37 [1.04-1.80] | < 0.05 |
| Stroke |  |  |  |  |  |  |
| No | ref |  |  | ref |  |  |
| Yes | 0.2 | 1.21 [0.92, 1.60] | 0.161 | -0.06 | 0.94 [0.70-1.28] | 0.709 |
| ^a^ SRH |  |  |  |  |  |  |
| Good | ref |  |  | ref |  |  |
| Poor | -0.2 | 0.82 [0.72, 0.94] | < 0.005 | -0.25 | 0.78 [0.68-0.90] | < 0.001 |
| Region |  |  |  |  |  |  |
| North | ref |  |  | ref |  |  |
| West | 0.07 | 1.08 [0.93 - 1.24] | 0.303 | -0.1 | 0.90 [0.77-1.07] | 0.238 |
| Northeast | -0.8 | 0.45 [0.37 - 0.53] | < 0.001 | -0.57 | 0.56 [0.46-0.70] | < 0.001 |
| East | -0.82 | 0.44 [0.38 - 0.50] | < 0.001 | -0.64 | 0.53 [0.45-0.62] | < 0.001 |
| Central | -0.8 | 0.46 [0.39 - 0.54] | < 0.001 | -0.52 | 0.59 [0.49-0.71] | < 0.001 |
| South | 0.29 | 1.33 [1.05 - 1.69] | <0.05 | 0.12 | 1.12 [0.94-1.35] | 0.215 |

**OR: Odd Ratio; CI: Confidence Interval; ref: reference; b: coefficient; ^a^ SRH: Self-rated health*
