# Supplementary material for: Socio-demographic and behavioral correlates of excess weight and its health consequences among older adults in India: Evidence from a cross-sectional study, 2017–18
Source: PLoS One. 2023 Oct 5;18(10):e0291920. doi: 10.1371/journal.pone.0291920 (PMC10553247; doi:10.1371/journal.pone.0291920)
Supplement: Attachment — Submitted filename: Response to Reviewer Comments.pdf [file pone.0291920.s003.pdf]

Response to the Editor comments

Thank you for the thorough review of our paper! We have made the changes as per the Editor's comments and used track changes to highlight the revisions within the manuscript. We provide an explanation below on how we address each comment provided in the edited copy of the manuscript. Kindly let us know if further changes are needed.

[p 2, line 41- 43]

The highest prevalence can be in one state, not in so many states. What is the rationale for choosing 33.2% prevalence cut-off? Authors can list out states above the national average.

The higher levels of excess weight (than the national average of 22.7%) were observed among older adults in states like Kerala, Karnataka, Himachal Pradesh, Punjab, and Sikkim………..has been updated in the revised manuscript.

[p 2, line 44- 45]

Replace respondents with older adults throughout the manuscripts, wherever the authors are presenting estimates. The estimates are presented for the study population, i.e., older adults.

Done! The word "older adults" is used throughout in the revised manuscript.

[p 2, line 47- 50]

Do the authors really want to highlight this association so explicitly? Education is correlated with the economic status of the population; this may not be the key factor in making someone overweight. Please consider the line of argument the authors want to put through their analysis and interpretation.

The statements are modified accordingly. They now read as follow:

Higher level of education is significantly positively correlated with excess weight. Similarly, higher household wealth index was significantly positively correlated with excess weight [OR: 1.77, CI: 1.46, 2.15].

[p 8, line 73- 75]

If the source of the information for these two sentences is the same, they can also be clubbed together, otherwise, the reference must be provided to the first sentence.

The statements are now clubbed together and the citation is provided at the end, and it reads as follows:

About 104 million people in India are 60 or older, constituting 8.6% of the total population, and by 2050, the percentage is expected to rise to 20% of the population.

[p 8, line 175- 177]

Authors are advised to reconsider the variables included in the health-related factors. Hypertension, Diabetes, stoke, and Heart diseases are not the determinants of obesity, but this is the other way around. These health issues are certainly associated with obesity, but they cannot be considered as its predictors. Authors can present these health issues as a consequence of obesity…

Done! The health-related variables are now removed from the predictors and a separate table is provided now to report the health-related consequences of excess weight among older adults.

[p 10, Table 1]

This should be replaced with MPCE Quintile

Done! The word "Wealth quintiles" is replaced by "MPCE quintiles" now.

[p 15, Table 3]

Combine the lower and upper bound of the 95% CI to present them in parentheses, e.g., (16.3, 18.9).

Present the numbers with single digit after decimal.

Thank you! The table is revised to incorporate both the suggestions.

[p 16, Table 3]

Also, provide the estimates for India (Total) in the last row.

Done!

[p 21, line 305-308]

This is the point the Authors have to recognize that these health effects are the resultant factor of overweight/obesity, not the determinants. So, present these associations in a separate section/table.

Done! In the revised manuscript, a separate table is provided now to report the health-related consequences of excess weight among older adults.

Response to the Reviewer comments

Reviewer #2: Thank you and well done for your much clearer revised manuscript.

Thank you for the thorough review of our paper! We have made the changes as per the Reviewer's comments and used track changes to highlight the revisions within the manuscript. Kindly let us know if further changes are needed.

Method
Line 39 - suggest to include definition of BMI 25 and above as excess weight.

[p – 37 -38]

Done! Now the definition has been provided with much more clarity.

Table 1:
- a few typos

Description in Consumption of tobacco - line 1 'redondents', line 5 'repond'

Done!

Description in Consumption of alcohol - line 2 'redondents'

Done!

Description Self-rated health - line 4 '...coded as bad' (I think it should be '..coded as poor')

Done!

Table 2 & 3 - please be consistent with decimal points.

Done!

[p 13, 15]
