# Supplementary material for: Socio-demographic and behavioral correlates of excess weight and its health consequences among older adults in India: Evidence from a cross-sectional study, 2017–18
Source: PLoS One. 2023 Oct 5;18(10):e0291920. doi: 10.1371/journal.pone.0291920 (PMC10553247; doi:10.1371/journal.pone.0291920)
Supplement: Attachment — Submitted filename: Response to Reviewer Comments.pdf [file pone.0291920.s004.pdf]

Response to the Editor comments

Thank you for the thorough review of our paper! We have made the changes as per the Editor's comments and used track changes to highlight the revisions within the manuscript. We address each comment in the revised manuscript. Kindly let us know if further changes are needed.

"The interpretation of Table 5 needs to be modified. The odds ratios of the logistic regression models suggest that older adults with excess weight were 2.19 times more likely to be hypertensive.... (similar interpretation in case of other diseases too), not the other way around as interpreted by the authors."

The interpretation of Table 5 is modified accordingly. They now read as follow:

"The odds ratios of the logistic regression models (Table 5) suggest that older adults with excess weight were 2.19 times [OR: 2.19, 95% CI: 1.92, 2.50], 2.14 times [OR: 2.14, 95% CI: 1.83, 2.50] and 1.63 times [OR: 1.63, 95% CI: 1.26, 2.11] significantly more likely to be hypertensive, diabetic and have heart disease. Similarly, older adults with excess weight were 0.85 times [OR: 0.85, 95% CI: 0.74, 0.98] significantly less likely to report good SRH in this study."

Authors are also requested to go through the entire manuscript again to ignore such mistakes, especially where the "health-related factors" were mentioned in the earlier manuscript and highlighted by the Editor. The health-related factors must be replaced with health consequences of excess weight wherever mentioned in the manuscript, and interpreted and discussed in similar lines.

Done!

We have gone through the whole manuscript and replaced the above-mentioned word in the entire manuscript!

Thank you for your time and support!
